# Comprehensive intellectual property ability and firm productivity: A quasi-natural experiment based on national intellectual property demonstration advantage firm policy in China

**DOI:** 10.1371/journal.pone.0302693

**Published:** 2024-04-25

**Authors:** Fang Li

**Affiliations:** School of Intellectual Property, Nanjing University Science & Technology, Nanjing, Jiangsu, China; Nanjing Audit University, CHINA

## Abstract

Intellectual property is crucial for the development of firms. At the micro level, firm comprehensive intellectual property ability involves abilities about intellectual property creation, utilization, protection, and management. In order to develop the comprehensive intellectual property ability of firms, the China National Intellectual Property Administration began to implement the national intellectual property demonstration advantage firm (NIPDAF) policy in 2013. Based on this exogenous policy shock, using data from listed companies from 2011 to 2020 as the research sample, the time-varying DID method is used to test the impact of the NIPDAF policy intended to cultivate comprehensive intellectual property ability on firm productivity. The results show that after policy implementation, the total factor productivity of NIPDAFs increased by about 3.3% compared to the control group. This finding is robust after a series of tests. Furthermore, the NIPDAF policy promotes firm productivity through stimulating technology innovation, improving investment efficiency, and enhancing competitive advantage. In addition, the NIPDAF policy has a more significant incentive effect on the total factor productivity of non-state-owned enterprises, firms in the eastern region, and firms in patent intensive industries.

## 1. Introduction

Intellectual property is the core element and ability of firm competitiveness [1]. With the advancement of networking and digitization, market competition and infringement disputes in the intellectual property are becoming increasingly incentivized. The development of firms is facing challenges, and higher requirements have been put forward for their comprehensive intellectual property ability. In recent years, China has attached increasing importance to intellectual property rights, accelerated the improvement of technology innovation level, and become the middle-income economy with the strongest innovation ability. According to the Global Innovation Index 2023 released by WIPO, China ranks 12th globally. However, the relationship between comprehensive intellectual property ability and firm productivity in existing research is not yet clear [2–5]. In this context, at the micro level, what role has the policy based on comprehensive intellectual property ability played in firm productivity?

Comprehensive intellectual property ability involves the creation, application, protection, and management of intellectual property, and is an important way for firms to improve their comprehensive strength. Meanwhile, intellectual property policy has a profound impact on regional and firm development [6, 7]. It is worth exploring whether policy makers can achieve the goal of improving firm productivity through intellectual property policy. In 2013, the China National Intellectual Property Administration (CNIPA) officially implemented the national intellectual property demonstration advantage firm (NIPDAF) policy, committed to improving the comprehensive intellectual property ability of firms. The NIPDAF policy is to promote the comprehensive development of firm intellectual property creation ability, application ability, protection ability, and management ability. The NIPDAF policy provides a series of policy benefits to NIPDAFs in R&D project, high-value patent cultivation, intellectual property financing, and administrative protection to increase comprehensive intellectual property ability. This policy promotes firm intellectual property creation and acquisition, market-oriented application, protection and maintenance, and strategic management, thereby improving productivity.

This paper constructs a panel data on productivity of Chinese listed companies from 2011 to 2020. Using the time-varying DID method, this study investigates whether the NIPDAF policy directly increases firm productivity through comprehensive intellectual property ability. At the same time, this study analyzes the mediating mechanisms of technology innovation, resource allocation efficiency, and market competitive advantage. In addition, this study further discusses the heterogeneity of policy effect from the perspectives of firm ownership, regional location, and industry type.

The main findings of this paper are as follows. (1) Compared with the control group firms, after the implementation of NIPDAF policy, the productivity of NIPDAFs significantly increased by about 3.3%. This provides empirical evidence for the direct promoting effect of comprehensive intellectual property ability on firm productivity. (2) After a series of robustness tests such as placebo test, Goodman-Bacon decomposition, PSM-DID method, replacement of dependent variable, and exclusion of contemporaneous policies, the conclusions still hold. (3) According to the analysis of impact mechanism, the NIPDAF policy indirectly increases firm productivity by promoting technology innovation, improving resource allocation efficiency, and enhancing market competitive advantage. (4) According to the results of heterogeneity analysis, the significant promoting effect of the NIPDAF policy on productivity is mainly reflected in non-state-owned enterprises, firms in the eastern region, and firms in patent intensive industries.

The innovation and research contributions of this paper are as follows. (1) Expanding Neves et al. [6] research on intellectual property, this study focuses on policy based on comprehensive intellectual property ability, and establishes a time-varying DID model to explore whether this intellectual property policy is effective for firm productivity. It enriches the connotation of intellectual property and provides a new understanding of the relationship between intellectual property and firm productivity. (2) This paper verifies the innovation incentive effect, resource allocation effect, and competitive advantage effect of NIPDAF policy on firm productivity. In addition to innovation, intellectual property can promote firm productivity by improving resource allocation efficiency and market competitiveness. It provides new insights into the indirect channels of intellectual property. (3) This paper considers the decisive role of firm ownership, regional location, and industry type in the effectiveness of NIPDAF policy. It reveals the differentiated characteristics of the effectiveness of NIPDAF policy. (4) Existing research on intellectual property policy evaluation mainly focuses on national and regional intellectual property policies. This paper fills the gap in the evaluation of intellectual property policy at the firm level.

The rest of the paper proceeds as follows. Section 2 provides a brief review of the existing studies. Section 3 presents the policy background and theoretical mechanism. Section 4 introduces the econometric model, variable selection and data description. Section 5 reports the results and analysis of empirical tests. Section 6 provides the conclusions and policy implications.

## 2. Literature review

The relevant literature of this study mainly involves two aspects: the impact of intellectual property on firm productivity and the evaluation of intellectual property policy.

In recent years, some studies have focused on the impact of intellectual property, especially intellectual property protection, on firm productivity. Intellectual property protection stimulates knowledge sharing and innovation output [8–10], promotes technology progress and productivity improvement in firms [11]. Simultaneously, intellectual property protection will also reduce knowledge spillovers [12–14], strengthen the technological and market monopoly position of firms, and reduce productivity. Therefore, empirical research has found inconsistent conclusions. Smeets and de Vaal [2] point out that intellectual property protection promotes knowledge sharing between multinational firms and local suppliers and improves supplier productivity, while increases the monopoly level of local customer firms, and reduces the productivity of local customer firms. Lai et al. [15] confirm that intellectual property enforcement reduces the productivity level of new technology enterprises, and higher enforcement levels will force low productivity enterprises to exit the market. Hu and Yin [5] find that strict intellectual property protection significantly improves firm productivity, and innovation activities and high-quality product imports are key mechanisms of effect.

In addition to intellectual property protection, of course, other intellectual property activities such as patent application and licensing can also affect firm productivity. Fang et al. [16] find that firm patent application behavior promotes productivity improvement, while local government patent subsidy policy weakens this promoting effect. Canavire-Bacarreza and Castro Peñarrieta [4] point out that intellectual property licensing policy affects the technology spillover effect of licensing and reduce firm productivity. He et al. [17] show that intellectual property rights based on innovation, such as patent and trademark, have a positive impact on firm productivity.

Regarding the research on intellectual property policy, existing literature mainly focuses on national and regional policies, such as intellectual property law [18–20], national intellectual property demonstration cities policy [21, 22], intellectual property subsidy policy [23, 24], patent priority review policy [25, 26], intellectual property pledge financing policy [27–29], and non-patent IP policies [30]. And existing literature analyzes the impact of these above intellectual property policies on technological innovation, knowledge dissemination, wage inequality, air pollution, etc. Individual studies have also explored the impact of intellectual property law on regional productivity. Sweet and Eterovic [31] point out that stricter patent law has no impact on productivity growth in developing and developed economies. It can be found that existing literature pays less attention to intellectual property policy at the firm level and overlooks the impact of intellectual property policy on firm productivity.

In summary, existing literature has discussed in detail about the relationship between intellectual property protection and firm productivity. However, a single dimension of protection cannot fully explain the impact of intellectual property on firm productivity. In addition to intellectual property protection, the creation, application, and management of intellectual property may also affect firm productivity. Therefore, the impact and mechanism of comprehensive intellectual property ability on firm productivity need further exploration. Moreover, existing literature mainly focuses on evaluating the effectiveness of regional and single dimensional intellectual property policy, with few studies focusing on firm level intellectual property policy and comprehensive intellectual property ability policy. To fill these gaps, this paper focuses on the NIPDAF policy aimed at developing comprehensive intellectual property ability, and explores its impact and mechanism on firm productivity.

## 3. Policy background and theoretical mechanism

### 3.1. Policy background

The NIPDAF policy is aimed at fostering the comprehensive intellectual property ability of firm. China launched the NIPDAF Policy in 2013. This policy is organized and implemented through independent application by firms and then evaluation by government. Government evaluates firm comprehensive intellectual property ability from the dimensions of intellectual property creation, utilization, protection, and management. Based on the evaluation results, the government determines the list of NIPDAFs. From 2013 to 2019, China has successively released six batches of NIPDAFs lists. This policy was not implemented in 2014. Affected by the COVID-19, the NIPDAF policy was forced to be interrupted in 2020 and 2021, and restarted in 2022.

**Fig 1:**
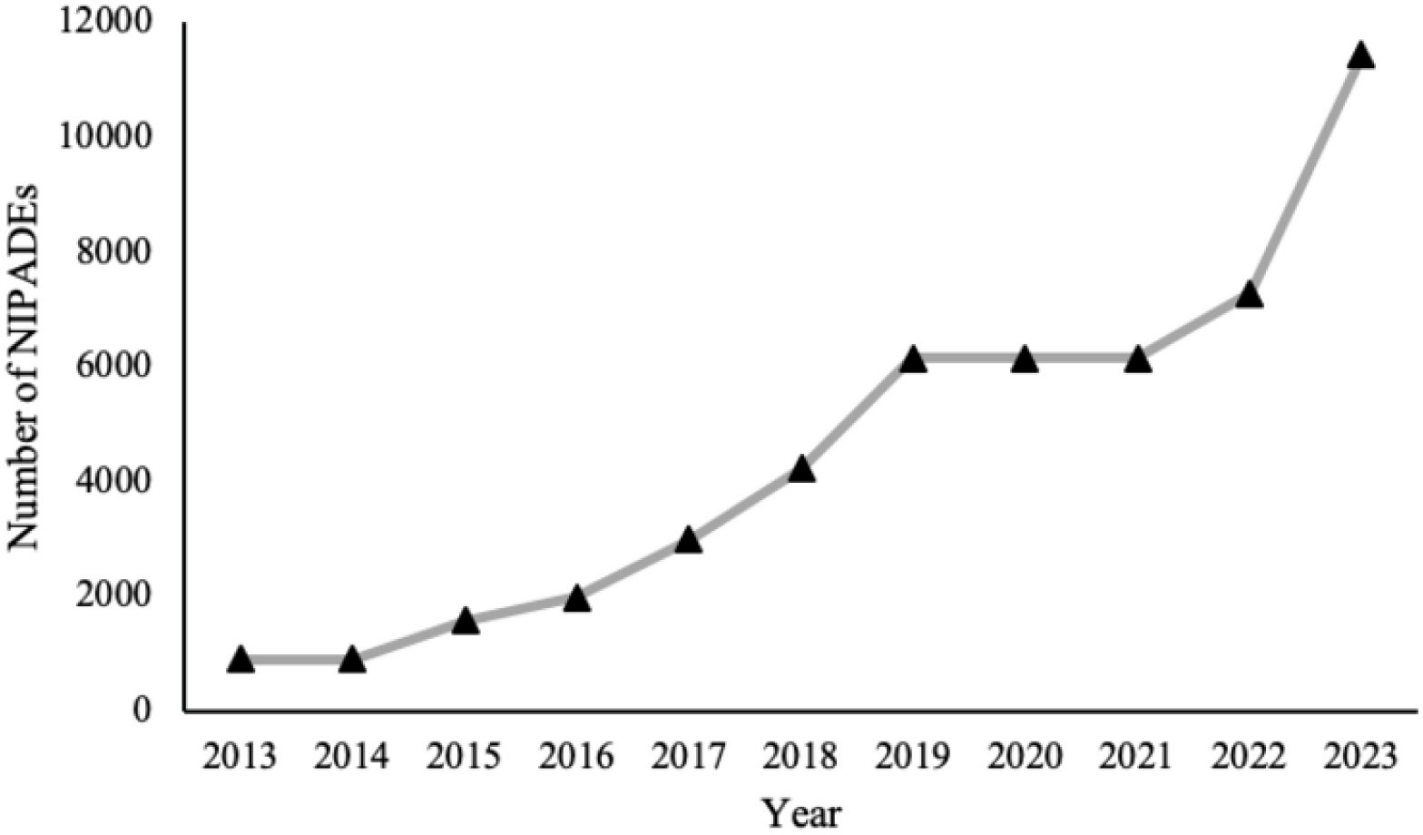
Number of NIPDAFs in China over the years since 2013.

The NIPDAF policy adopts a dynamic evaluation mechanism. An assessment period is 3 years, and a re-evaluation will be conducted at the end of the period. Firms that pass the re-evaluation can continue to retain their qualifications. If a firm fails to re-evaluation, the qualification of the firm will be cancelled. The number of NIPDAFs in China over the years since 2013 is shown in Fig 1. As of the end of 2019, there were a total of 6145 NIPDAFs within the validity period. In 2023, the number of NIPDAFs reached 11426.

The government provides a series of preferential policies to NIPDAFs. In addition to honorary rewards, the government also provides support in areas such as technology innovation, intellectual property talent cultivation, industrial cooperation, and financial subsidy, and so on. For example, the government will prioritize supporting NIPDAFs in carrying out patent navigation projects and assisting NIPDAFs in training intellectual property management talents. These supportive measures can help to enhance the comprehensive intellectual property ability of NIPDAFs.

### 3.2. Theoretical mechanism and research hypothesis

#### 3.2.1 Comprehensive intellectual property ability and firm productivity

Comprehensive intellectual property ability represents the strength of intellectual property creation, application, protection, management. As a policy dedicated to cultivating comprehensive intellectual property ability, the NIPDAF policy may promote firm productivity in four ways. Firstly, intellectual property creation is closely related to firm productivity. Intellectual property creation not only requires innovation, but also requires firms to choose to protect innovation in the form of intellectual property [32, 33]. The NIPDAF policy encourages firms to formally protect innovation through intellectual property rights. After the implementation of NIPDAF policy, firms are more willing to increase investment in intellectual property application and maintenance, create more and higher quality intellectual property, and thereby improve total factor productivity. Secondly, intellectual property application is the core way for firm to transform new technology into advanced productivity. By transforming and applying new technology and new design represented by intellectual property, firms can improve production processes to reduce costs, and develop new products to obtain innovative benefits [34, 35]. The NIPDAF policy expands the ways in which intellectual property value can be realized. In addition to self-use, firms can explore various application methods such as intellectual property transfer, licensing, pledge financing, etc. The NIPDAF policy facilitates intellectual property application to promote firm technology progress, thereby improving total factor productivity. Thirdly, intellectual property protection provides necessary support for stimulating innovation and obtaining innovative rent. Intellectual property protection can reduce the risk of spillover of new knowledge and technology [36, 37], as well as the uncertainty of informal protection, and avoid the negative impact of technology spillover on productivity. The NIPDAF policy provides assistance in intellectual property disputes and enhances firm intellectual property protection ability. The NIPDAF policy guides firms to strengthen intellectual property protection and maintain intellectual property advantages, and thereby promote productivity. Fourthly, intellectual property management is an important part of firm strategic management. Based on the business objectives of the firm, intellectual property strategic management clarifies the development direction and route of intellectual property, and improves the efficiency of business management [38, 39]. The NIPDAF policy propels firms to perfect intellectual property management system and establish specialized intellectual property management institutions and teams. The NIPDAF policy raises firm productivity by comprehensively enhancing the ability to create, apply, protect, and manage intellectual property. Based on above-mentioned, hypothesis 1 is proposed.

H1: The NIPDAF policy significantly increases firm total factor productivity.

#### 3.2.2 Impact mechanism

The NIPDAF policy can promote firm productivity through stimulating innovation mechanism. Intellectual property plays a significant role in promoting innovation [6, 10]. The NIPDAF policy prioritizes supporting firm to undertake government science and technology project and encourages firm to increase R&D investment. The government also prioritizes assisting firm in conducting industry university research cooperation, which can accelerate technology innovation and intellectual property creation. Intellectual property protection assures innovation returns and motivates firm to continuously carry out innovation activities. Innovation is the core element that affects firm productivity [40, 41]. Especially, substantive innovation represented by high-quality patent can evidently spurring firm technical advancement. By adopting new technology and novation, firms can improve production processes and management efficiency, and then increase productivity. Based on the above, hypothesis 2 is proposed.

H2: The NIPDAF policy increases firm productivity by promoting technology innovation.

The NIPDAF policy can promote firm productivity through resource allocation effect. The NIPDAF policy enriches firm intellectual property resources and other production resources by strengthening intellectual property creation, utilization, and protection. For example, intellectual property pledge financing can expand firm financing channels and increase funding supply. The NIPDAF policy guides firm to allocate production factors around intellectual property. This helps to deepen the organic integration of intellectual property resources with technology, capital, labor and other production resources. New resource allocation way avoids the problems of inefficient allocation and resource mismatch [42]. Resource allocation is another key factor affecting firm productivity [43, 44]. Effective resource allocation enables optimal utilization of various production resources, including intellectual property resources. Conversely, inefficient resource allocation will lead to resource waste and result in losses of firm productivity. Optimizing resource allocation efficiency is an effective way to improve firm productivity. Based on the above, hypothesis 3 is proposed.

H3: The NIPDAF policy increases firm productivity by improving resource allocation efficiency.

The NIPDAF policy can promote firm productivity through competitive advantage effect. Comprehensive intellectual property ability will enhance firm core competitiveness and enable to quickly gain market advantage. The NIPDAF policy drives firm to develop new product, explore new market, and increase market share. Intellectual property protection prevents competitors from imitating and maintains the technology and market advantages of firms. The NIPDAF policy enhances the market competitive advantage of firms. Market competitive advantage reduces operational costs, improves performance, and promotes productivity improvement [45, 46]. At the same time, market competitive advantage alleviates firm financial constraint, increase investment in innovation and intellectual property, and thereby improve productivity. Based on the above, hypothesis 4 is proposed.

The H4: The NIPDAF policy increases firm productivity by enhancing market competitive advantage.

**Fig 2:**
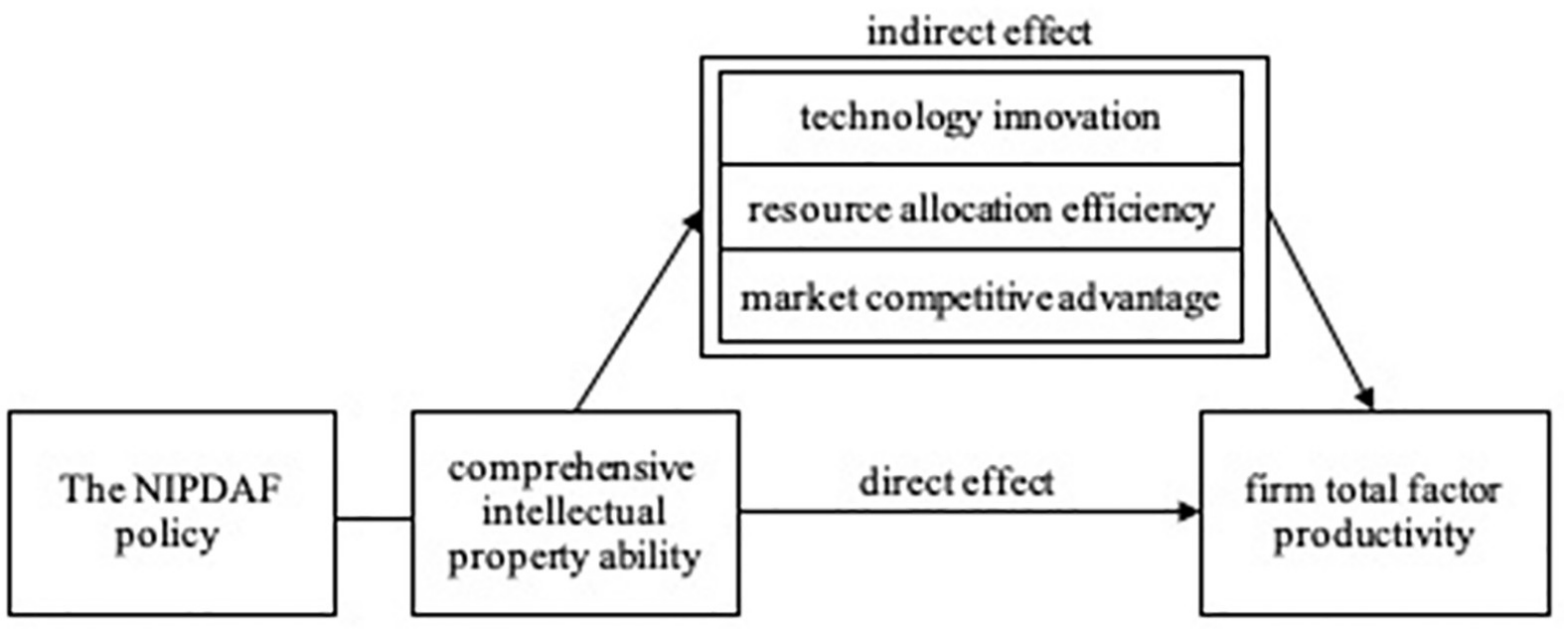
Impact mechanism of comprehensive intellectual property ability on firm productivity.

Fig 2 shows the impact mechanism of comprehensive intellectual property ability on firm productivity.

## 4. Methodology and data

### 4.1. Econometric model

This paper regards the NIPDAF policy in China as a quasi-natural experiment. Based on the panel data of listed companies in China from 2011 to 2020, the NIPDAFs are treated as the treatment group, and the other listed firms are treated as the control group. Due to the NIPDAF policy is implemented in batches in different years, this study constructs a time varying DID model as follows.


$$Y_{it}=\alpha_{0}+\alpha_{1}{did}_{it}+\alpha_{2}X_{it}+\mu_{i}+\theta_{s}+\lambda_{t}+\varepsilon_{it}$$ (1)

In the above equation, *Y*_*it*_ represents the total factor productivity of firm *i* in year *t*; *did*_*it*_ is the dummy variable of whether firm *i* is a NIPDAF in year *t*; *X*_*it*_ is the collection of control variables; *μ*_*i*_ represents the firm fixed effect; *θ*_*s*_ represents the industry fixed effect; *λ*_*t*_ represents the time fixed effect; *ε*_*it*_ represents the random error term. If *α*_1_ is significantly greater than 0, it indicates that the NIPDAF policy has a significant promoting effect on firm productivity.

On the basis of evaluating the direct impact, this paper uses the mediation effect model to test the indirect impact of the NIPDAF policy on firm productivity. Including Eq (1), the remaining two steps of the mediation effect model are as follows.


$$M_{it}=\beta_{0}+\beta_{1}{did}_{it}+\beta_{2}X_{it}+\mu_{i}+\theta_{s}+\lambda_{t}+\varepsilon_{it}$$ (2)

$$Y_{it}=\gamma_{0}+\gamma_{1}{did}_{it}+{\gamma_{2}M}_{it}+\gamma_{3}X_{it}+\mu_{i}+\theta_{s}+\lambda_{t}+\varepsilon_{it}$$ (3)

In the above equation, *M*_*it*_ represents the collection of mediation variables, including technological innovation, resource allocation efficiency, and market competitive advantage; the other variables are the same as Eq (1). *β*_1_ and *γ*_2_ are the coefficients that this mediation effect model focuses on. If *β*_1_ and *γ*_2_ are both significantly greater than 0, it indicates that the intermediary is an important mechanism for the NIPDAF policy to promote firm productivity.

### 4.2. Variable selection

Dependent variable (*TFP*). Total factor productivity (*TFP*) is the comprehensive allocation efficiency of various factor resources, can more comprehensively and systematically display the productivity level of firm [3, 5]. Following Fang et al. [3], Hu and Yin [5] and Zhang et al. [47], this study uses the total factor productivity calculated by LP method for baseline regression, and uses the total factor productivity calculated by OP method, GMM method, and CFA method for robustness test.

Independent variable (*DID*). *DID* is the dummy variable of NIPDAF policy. A validity period of the NIPDAF is 3 years. If the firm fails to pass the review upon the expiration of the validity period, the qualification of NIPDAF will be cancelled. Therefore, if an authenticated firm fails the review, it will be assigned as 0 in the fourth year of authentication and thereafter. If a NIPDAF passes the review or upgrades from a NIPAF to a NIPDF, it will continue to be assigned as 1 for the following three years.

Mediating variables. In order to test the indirect effect, this paper constructs three mediating variables.

Technology innovation (*TI*). Patent application can reflect firm technology innovation ability timely and reliably. Following Dai and Sun [44] and Zhang et al. [26], the number of patent application is used to measure the level of firm technology innovation.

Resource allocation efficiency (*RAE*). *RAE* is measured by firm investment efficiency. Referring to Richardson [48], the following model is used to estimate firm investment efficiency.


$${Invest}_{it}=\rho_{0}+\rho_{1}{Invest}_{it-1}+\rho_{2}{lev}_{it-1}+\rho_{3}{roa}_{it-1}+\rho_{4}{age}_{it-1}+\rho_{5}{ocf}_{it-1}+\rho_{6}{size}_{it-1}+\rho_{7}{tobin}_{it-1}+\rho_{8}{grow}_{it-1}+\rho_{9}{ret}_{it-1}+\mu_{i}+\theta_{s}+\lambda_{t}+\varepsilon_{it}$$ (4)

In the above equation, *Invest*_*it*_ represents firm investment level, measured by the proportion of the original price of fixed assets to the total assets; *Invest*_*it*−1_ is the investment level that lags behind one period; *lev*_*it*−1_ is firm asset liability ratio, measured by the ratio of total liabilities to total assets; *roa*_*it*−1_ is the return on assets; *age*_*it*−1_ is firm age; *ocf*_*it*−1_ is the ratio of cash flow from operating activities to total assets; *size*_*it*−1_ is firm size, measured by the logarithm of the total assets; *tobin*_*it*−1_ is the investment opportunity, measured by Tobin’s Q value; *grow*_*it*−1_ is the growth rate of operating revenue; *ret*_*it*−1_ represents the earnings per share of the firm. Residual value *ε*_*it*_ represents the degree of firm investment inefficiency. *RAE = |ε*_*it*_|. *RAE* is a reverse measurement indicator, that is, the larger the value of *RAE*, the lower the firm resource allocation efficiency.

Market competitive advantage (*MCA*). Following Peress [49], the Lerner index is used to measure product market competitive advantage of the firm. The larger the value of *MCA*, the stronger the pricing ability of the firm in the product market, and the more significant the market competitive advantage.

Control variables. Considering other factors that may affect firm productivity, and following He et al. [17], and Kong et al. [45], this paper selects ten control variables. The scale of the enterprise (*size*), profitability (*roa*), development ability (*grow*) and debt paying ability (*lev*) are the same as Eq (4). Other control variables include labor input (*labor*), corporate governance ability (*board*), corporate governance structure (*idr*), government subsidy (*g_fund*), nature of property rights (*soe*), and industry concentration (*hhi*).

The description and measurement for the variables are shown in Table 1.

**Table 1:** Description and measurement for the variables.

	Variable	Symbol	Measurement
Dependent variable	Total factor productivity	*TFP*	calculated by LP method
Independent variable	Comprehensive intellectual property ability	*DID*	dummy variable, if and only if firm *i* is a NIPDAF in year *t*, it is assigned a value of 1; otherwise, it is 0
Mediating variable	Technology innovation	*TI*	natural logarithm of the total number of patent application+1
	Resource allocation efficiency	*RAE*	Referring to Richardson [48]
	Market competitive advantage	*MCA*	(operating revenue—operating costs—sales expenses—management expenses)/operating revenue
Control variable	Scale of the enterprise	*size*	natural logarithm of the total assets
	profitability	*roa*	the return on assets
	development ability	*grow*	growth rate of operating revenue
	debt paying ability	*lev*	the ratio of total liabilities to total assets
	Labor input	*labor*	natural logarithm of the number of employees
	Corporate governance ability	*board*	natural logarithm of the number of board members
	Corporate governance structure	*idr*	the rate of independent directors in the board of directors
	Government subsidy	*g_fund*	government funds received by enterprises
	nature of property rights	*soe*	dummy variable, when the firm is a state-owned enterprise, it is recorded as 1, otherwise it is 0
	Industry concentration	*hhi*	the sum of the squares of the rate of the main business income of each firm on the total income in the industry

### 4.3. Data

This paper uses the panel data of 1742 Chinese A-share listed companies from 2011 to 2020. Due to the impact of the COVID-19, China did not implement this policy in 2020 and 2021. Therefore, this article chooses 2011–2020 as the research period. The data of the implementation years and the firm lists of NIPDAF policy is obtained from the official website of China National Intellectual Property Administration (https://www.cnipa.gov.cn/). The original data of other variables is obtained from the CSMAR database.

The original data was processed as follows: excluding financial companies, ST companies, severely missing data, and abnormal data samples. In order to avoid the influence of extreme outliers, continuous variables are subjected to 1% and 99% percentile truncation. Finally, 17420 sample observations from 1742 listed companies were retained.

Descriptive statistics for the variables are reported in Table 2. The maximum and minimum values of *TFP* are 12.072 and 2.531, respectively, indicating a significant difference in productivity among the sample firms. The mean *DID* is 0.118, indicating that approximately 11.8% of the sample observations are affected by the NIPDAF policy. The data distribution of other variables shows no significant skewness.

**Table 2:** Descriptive statistics for the variables.

	Variable	N	Mean	Standard deviation	Max	Median	Min
Dependent variable	*TFP*	17420	4.980	0.390	12.072	4.962	2.531
Independent variable	*DID*	17420	0.118	0.323	1	0	0
Mediating variable	*TI*	17420	1.768	1.761	9.821	1.609	0
	*RAE*	17420	0.032	0.038	0.484	0.020	0
	*MCA*	17420	0.123	0.129	0.847	0.104	-2.754
Control variable	*size*	17420	22.413	1.311	25.944	22.244	19.373
	*roa*	17420	0.038	0.052	0.197	0.035	-0.242
	*grow*	17420	0.066	0.266	0.874	0.088	-1.322
	*lev*	17420	0.432	0.203	0.887	0.431	0.053
	*labor*	17420	7.856	1.251	10.963	7.801	4.234
	*board*	17420	0.374	0.057	0.800	0.333	0.167
	*idr*	17420	2.145	0.199	2.890	2.197	1.099
	*g_fund*	17420	12.481	6.697	20.131	15.401	0
	*soe*	17420	0.431	0.495	1	0	0
	*hhi*	17420	0.121	0.128	1	0.081	0.013

**Fig 3:**
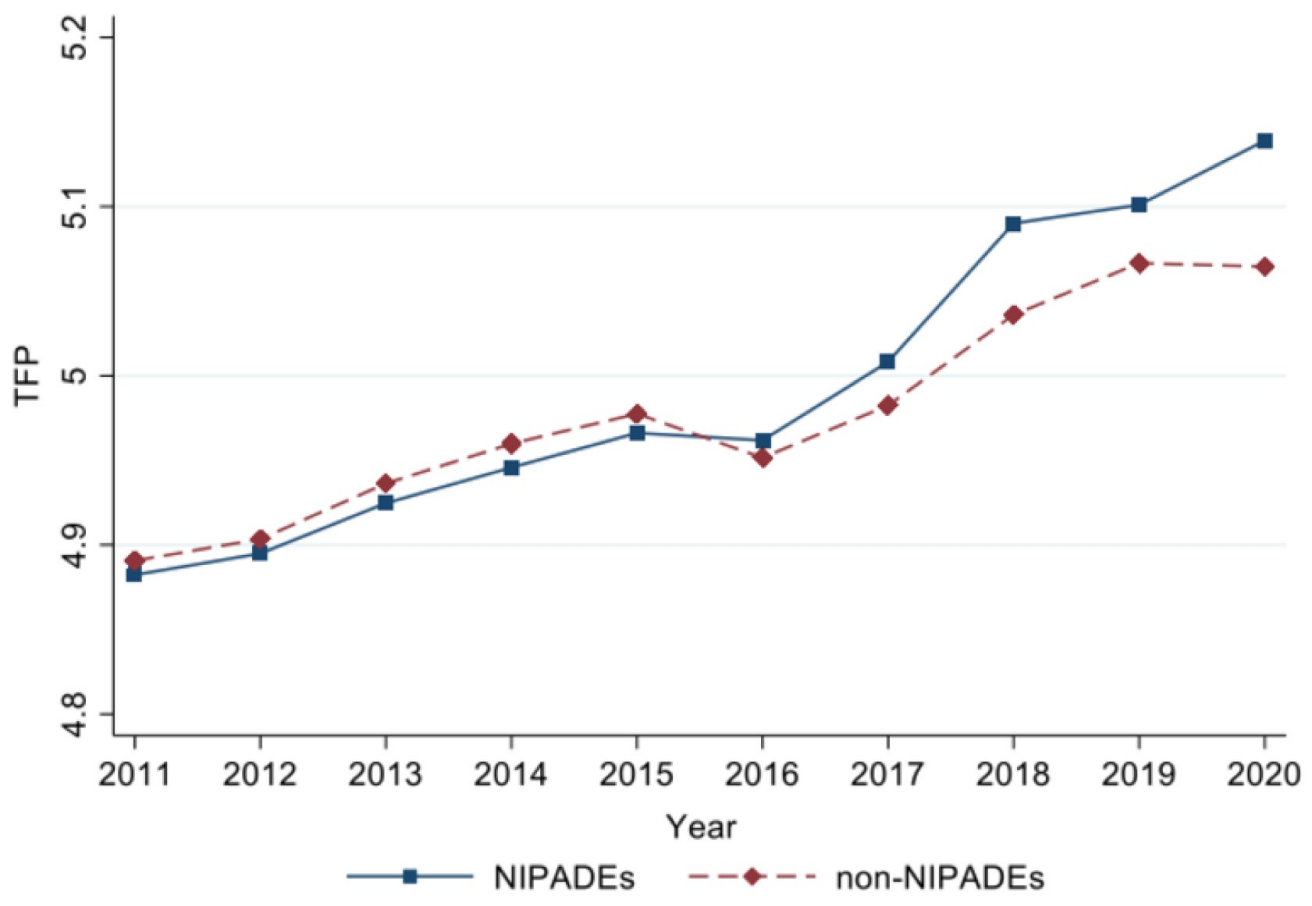
Trend of firm productivity in 2011–2020.

Fig 3 shows the average trend of productivity of firms in the treatment group and control group. Before the implementation of the NIPDAF policy, the firm productivity of the treatment group and the control group maintained the same trend. After the implementation of the policy, the firm productivity of the treatment group showed a significant improvement trend, with a growth rate obviously higher than that of the control group. The parallel trend hypothesis has been preliminarily validated.

## 5. Empirical results

### 5.1. Basic model regression results

Baseline regression is conducted based on Eq (1), and the estimation results are shown in Table 3. Column (1) and (2) show the regression results of the NIPDAF policy on *TFP* without control variables. Column (3) presents the estimated results including control variables, and column (4) presents the estimated results including control variables and fixed effects. The estimated coefficients of *DID* in columns (1)-(4) are all positive and significant at the 1% level, indicating that the NIPDAF policy has a significant promoting effect on firm productivity. The coefficient of *DID* in column (4) is 0.033, which means that the NIPDAF policy has increased firm total factor productivity by 3.3% compared to the control group. The NIPDAF policy has improved firm comprehensive intellectual property ability, promoted intellectual property creation, strengthened intellectual property protection, promoted intellectual property utilization, improved management efficiency, and ultimately improved total factor productivity. Therefore, the NIPDAF policy based on comprehensive intellectual property ability has significantly improved firm productivity, and this result supports H1.

**Table 3:** Results of basic model regression.

Variable	(1)	(2)	(3)	(4)
*DID*	0.150***	0.047***	0.037***	0.033***
	(0.009)	(0.009)	(0.008)	(0.008)
*size*			0.177***	0.164***
			(0.005)	(0.006)
*roa*			1.027***	1.061***
			(0.043)	(0.043)
*grow*			0.100***	0.120***
			(0.007)	(0.007)
*lev*			0.077***	0.067***
			(0.019)	(0.020)
*labor*			-0.023***	-0.020***
			(0.005)	(0.005)
*board*			-0.001	0.003
			(0.019)	(0.019)
*idr*			-0.102*	-0.106**
			(0.054)	(0.054)
*g_fund*			-0.016***	0.003
			(0.003)	(0.004)
*soe*			0.005	-0.006
			(0.014)	(0.014)
*hhi*			0.018	-0.020
			(0.025)	(0.031)
Constant	4.962***	4.974***	1.156***	1.425***
	(0.002)	(0.002)	(0.099)	(0.120)
*N*	17420	17420	17420	17420
Enterprise FE	Yes	Yes	Yes	Yes
Industry FE	No	Yes	No	Yes
Year FE	No	Yes	No	Yes
adj. *R*^2^	0.655	0.687	0.727	0.736

Note:

***, **, and * indicate the significance at 1%, 5%, and 10% levels, respectively; standard errors in parentheses. YES means the fixed effect is controlled.

### 5.2. Robustness test

#### 5.2.1. Parallel trend test

Parallel trend assumption means without the implementation of NIPDAF policy, firm productivity in the two groups should maintain the same trend. Fig 3 provides rough evidence. In order to further enhance reliability, the event study method is used for parallel trend test.

In the time-varying DID model with heterogeneous treatment effects, negative weights may affect the effectiveness of the model and even lead to a reverse effect [50]. Borusyak et al. [51] propose a new DID estimation based on interpolation method, which applies with time-varying controls. Therefore, using the method proposed by Borusyak et al. [51], the dynamic treatment effects are estimated according to the following model.


$$Y_{it}=\alpha_{0}+\alpha_{1}{did}_{it}+\alpha_{2}X_{it}+\mu_{i}+\theta_{s}+\lambda_{t}+\varepsilon_{it}$$ (5)

**Fig 4:**
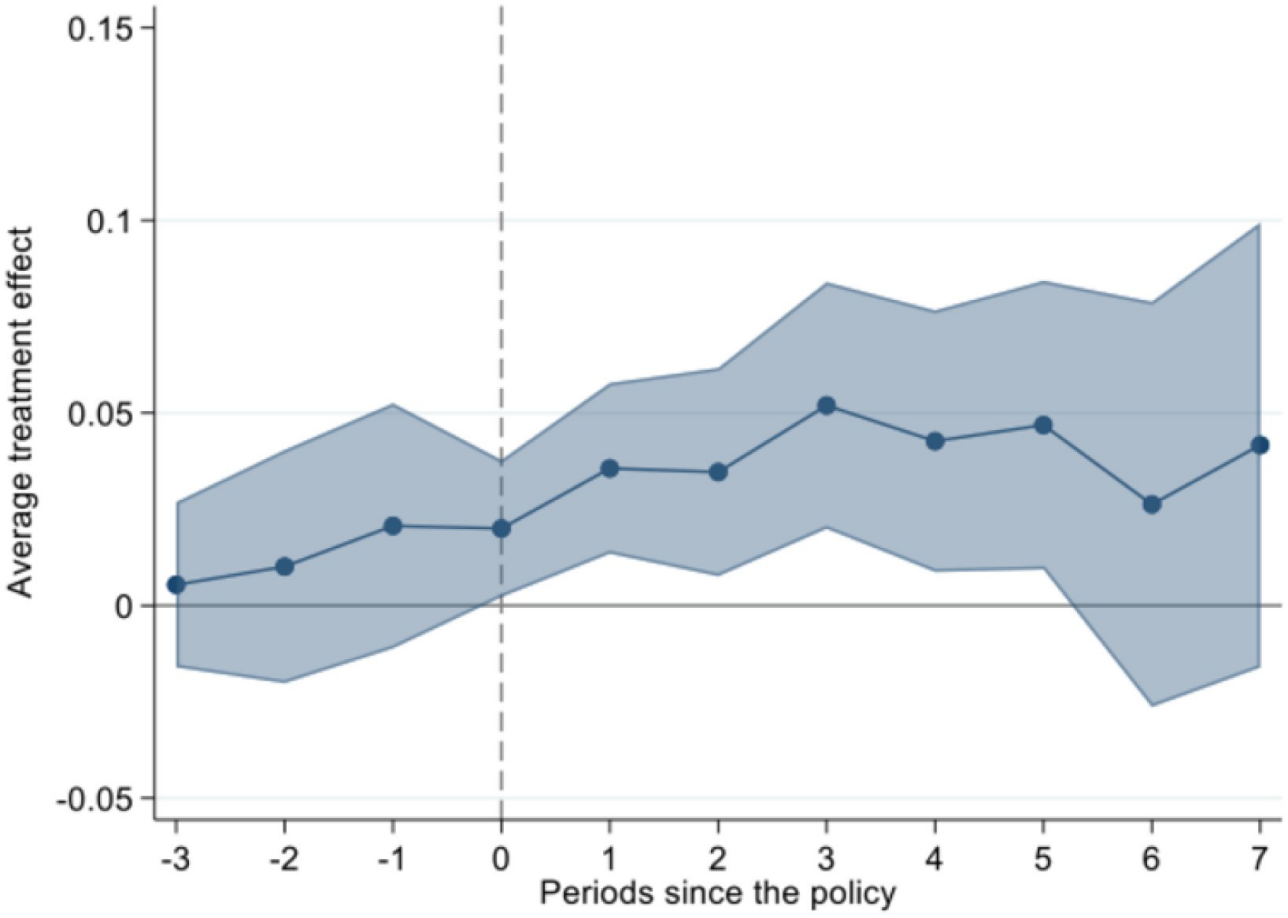
Parallel trend test.

Fig 4 shows the results of parallel trend test. It can be seen that before the implementation of the NIPDAF policy, the trend of productivity in the treatment group and the control group are basically the same, meeting the parallel trend assumption. Within 5 years after policy implementation, the estimated coefficients are significantly positive, which shows different trends of productivity between the two groups. Comparing to non-NIPDAFs companies, the productivity of NIPDAFs has significantly increased.

#### 5.2.2. Goodman-Bacon decomposition

Under the bidirectional fixed effect, the variation in treatment groups and treatment times will result in heterogeneous treatment effects and biased DID estimators [52]. To analysis the degree of bias caused by heterogeneous treatment effects, the baseline model was tested by the estimator decomposition method proposed by Goodman-Bancon [53]. Table 4 shows the results of Goodman-Bacon decomposition method. It can be seen that the weight of the inappropriate treatment effect, Late Treatment vs. Early Comparison, is only 4.2%, while the weight of the appropriate treatment effect is 95.8%. This indicates that the majority of the treatment effect in baseline estimation come from the analysis of the treatment firms and the firms have never been treated. Therefore, the results of baseline regression are reliable, and the problem of heterogeneity treatment effect has not caused serious estimation bias.

**Table 4:** Goodman-Bacon decomposition.

DID decomposition comparison	weight	coefficient of *DID*
Earlier Treatment vs. Later Comparison	0.042	0.012
Later Treatment vs. Earlier Comparison	0.052	0.034
Treatment vs. Never treated	0.906	0.048

#### 5.2.3. Placebo test

**Fig 5:**
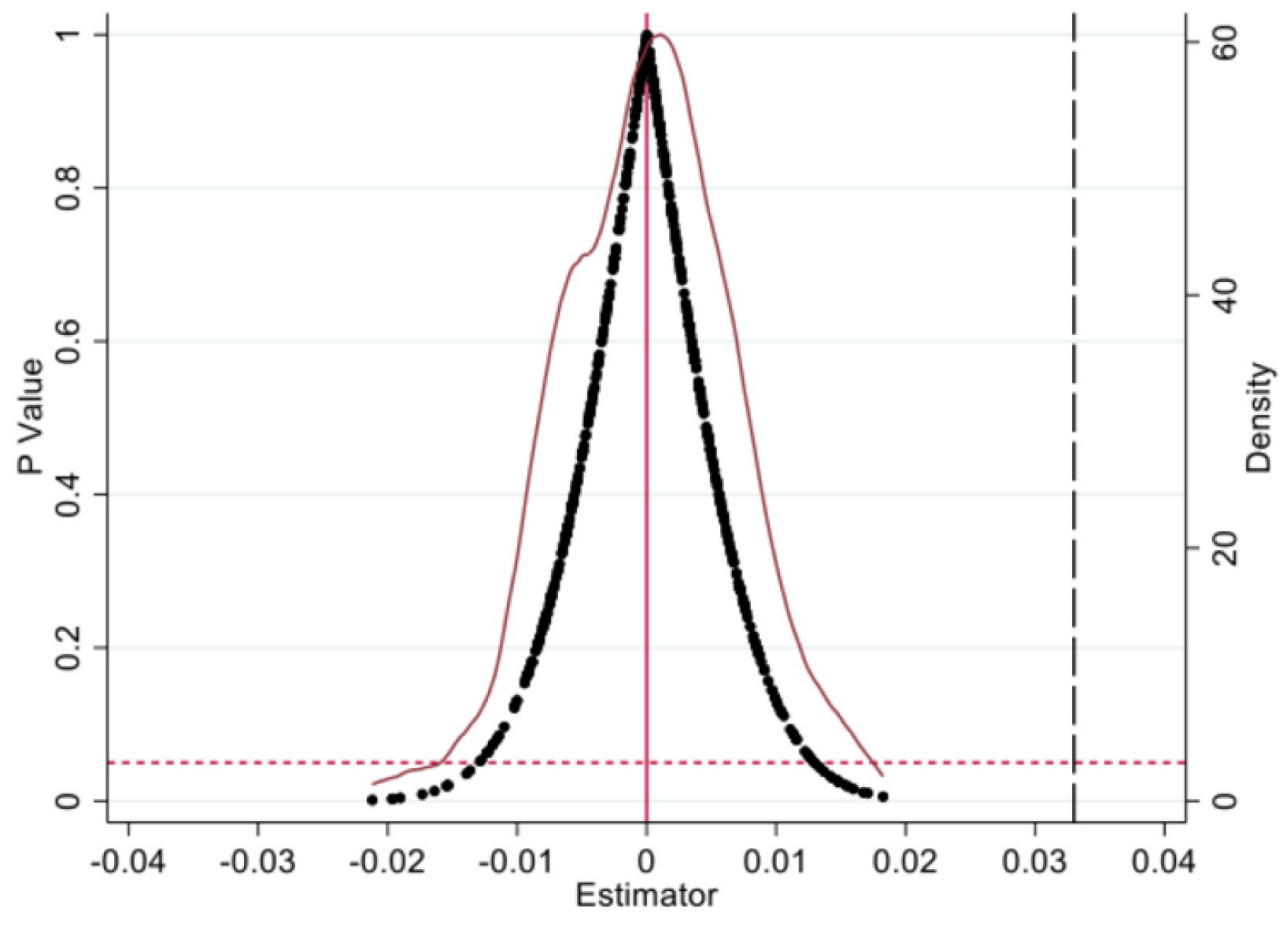
Placebo test.

To avoid the impact of random effects or other unknown factors on firm productivity, it is necessary to conduct placebo test. Randomly select the experimental group and policy time from all samples, and then perform baseline regression. After 500 rounds of sampling and regression, the coefficient p-value and kernel density distribution are shown in Fig 5.

It can be seen that the estimated coefficients approximately follow a normal distribution, with coefficient values clustered around 0. The majority of estimated coefficients in sampling regression have p-values above 0.1. It indicates that policies generated by random sampling have no significant impact on firm productivity. Meanwhile, there is a significant difference between the coefficient of baseline estimation and the estimation coefficients of placebo test. It means that the impact of random effects or unobservable factors on firm productivity can be excluded. Therefore, it can be concluded that the increase in firm productivity is caused by the NIPDAF policy.

#### 5.2.4. PSM-DID

Considering the application and identification of NIPDAF is not completely random, that may have the problem of selection bias. Propensity score matching (PSM) method can effectively alleviate selection bias and improve the accuracy of DID estimation. Following Lv et al. [21] and Zhang et al. [26], this paper uses the PSM-DID method for robustness test.

Firstly, the Logit model is used to estimate the propensity score value with covariates. Considering factors that may affect whether a firm becomes a NIPDAF, this paper selects firm characteristic variables such as *TI*, *labor*, *roa*, *grow*, *Invest*, *board*, *size*, *age*, *Iev* and *soe* as covariates.

**Fig 6:**
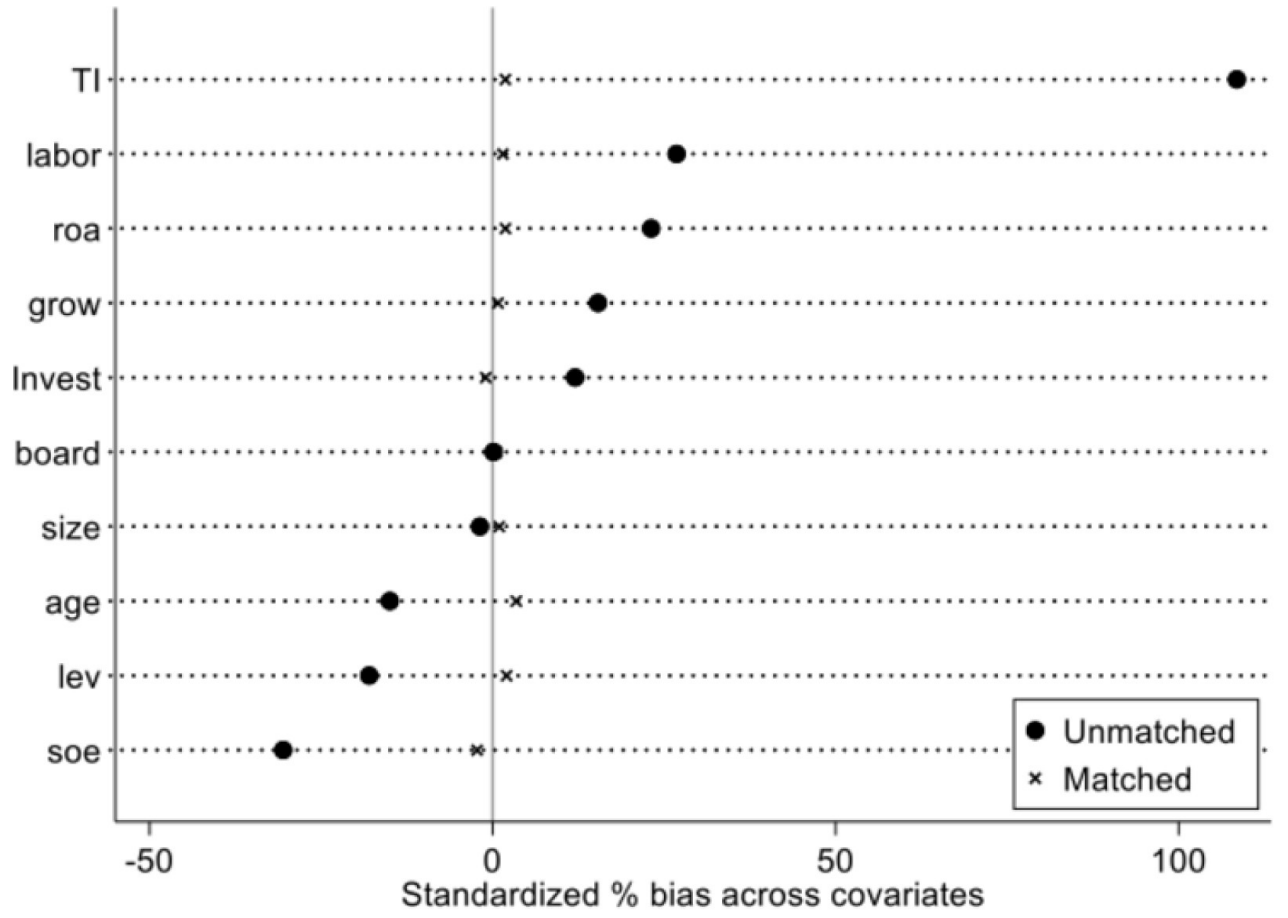
Results of balance hypothesis test.

Secondly, the nearest neighbor matching method within the caliper is used for sample data matching. Matched samples are obtained through annual matching. The results of balance hypothesis test after PSM are shown in Fig 6. It can be seen that the standard deviation of all covariates has significantly decreased. There is no significant difference in covariates between the treatment group and the matched control group. The matching results balance the data well.

**Fig 7:**
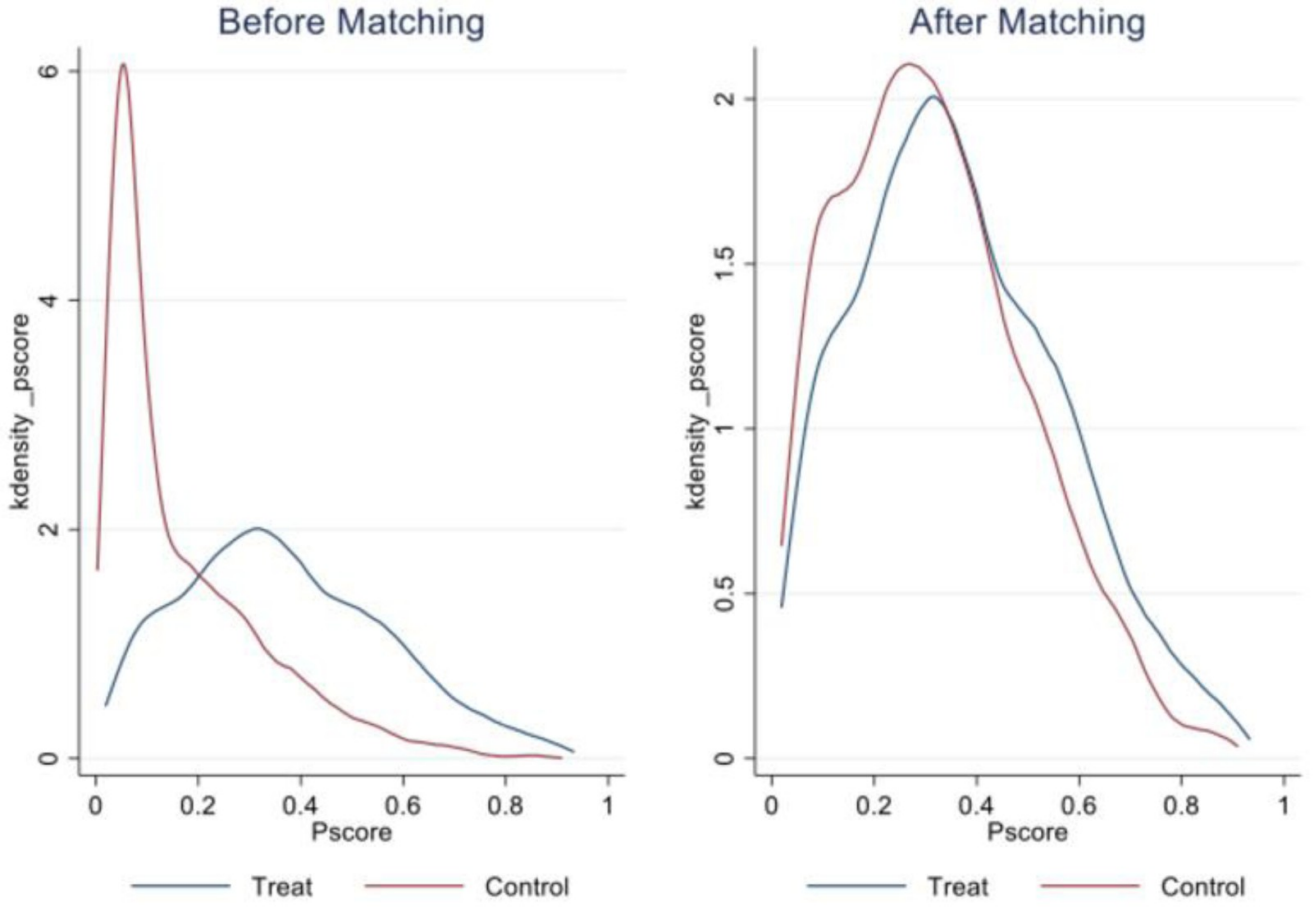
Kernel density plot of propensity score values before and after PSM.

Thirdly, draw the kernel density plot of propensity matching scores. If the kernel density curves between the treatment group and the control group have a significant deviation before matching, while the kernel density curves after matching are relatively close, it indicates that PSM has a good matching effect. Fig 7 shows the kernel density plot of propensity score values before and after PSM. It can be seen that PSM matching has achieved significant results. Therefore, to some extent, PSM method has a treatment effect on reducing sample selectivity bias.

Fourthly, based on the PSM matched data, the DID method is used for regression estimation. Table 5 column (1) and (2) show the estimated results of PSM-DID. Column (1) displays the estimation results based on samples that satisfy the common support assumption. Column (2) displays the estimation results based on samples with non-empty weight. The coefficients of PSM-DID estimation are less different from the coefficient of baseline regression, and both are significant at the 1% level. Therefore, the results of baseline regression are robust.

**Table 5:** Results of PSM-DID and alternative indicators.

	(1)	(2)	(3)	(4)	(5)
	on_support	weight	TFP_op	TFP_gmm	TFP_cfa
*DID*	0.029***	0.027***	0.028***	0.033***	0.031***
	(0.008)	(0.010)	(0.011)	(0.008)	(0.008)
*size*	0.160***	0.192***	0.532***	0.203***	0.351***
	(0.006)	(0.010)	(0.008)	(0.006)	(0.006)
*roa*	1.111***	1.220***	1.196***	1.064***	1.122***
	(0.044)	(0.083)	(0.057)	(0.041)	(0.042)
*grow*	0.110***	0.057***	0.365***	0.141***	0.240***
	(0.007)	(0.015)	(0.009)	(0.006)	(0.007)
*lev*	0.085***	0.180***	0.454***	0.105***	0.264***
	(0.020)	(0.038)	(0.026)	(0.019)	(0.019)
*labor*	-0.017***	-0.045***	0.062***	-0.053***	-0.012**
	(0.005)	(0.012)	(0.007)	(0.005)	(0.005)
*board*	-0.133**	0.013	0.148**	-0.083	0.017
	(0.055)	(0.103)	(0.071)	(0.052)	(0.052)
*idr*	-0.007	0.057	0.055**	0.007	0.028
	(0.019)	(0.035)	(0.025)	(0.018)	(0.018)
*g_fund*	0.004	-0.017***	0.002	0.002	0.003
	(0.004)	(0.005)	(0.005)	(0.004)	(0.004)
*soe*	-0.008	0.039	0.010	-0.006	0.001
	(0.014)	(0.026)	(0.018)	(0.013)	(0.013)
*hhi*	0.009	0.060	-0.046	-0.026	-0.042
	(0.032)	(0.068)	(0.041)	(0.030)	(0.030)
Constant	1.494***	0.789***	3.901***	1.713***	2.725***
	(0.122)	(0.202)	(0.160)	(0.116)	(0.117)
*N*	16528	4708	17420	17420	17420
Enterprise FE	Yes	Yes	Yes	Yes	Yes
Industry FE	Yes	Yes	Yes	Yes	Yes
Year FE	Yes	Yes	Yes	Yes	Yes
adj. *R*^2^	0.735	0.764	0.944	0.786	0.923

#### 5.2.5. Alternative indicators of the dependent variable

To ensure robustness, OP method, GMM method, and CFA method are used instead of LP method to calculate total factor productivity as alternative indicators of the dependent variable (*TFP*). Using Eq (1) to perform baseline regression again, columns (3)-(5) in Table 5 report the regression results after using alternative variables. It can be seen that the coefficients of *DID* are significantly positive and similar to the estimated coefficient of the baseline regression. The incentive effect of the NIPDAF policy on firm productivity does not depend on the measurement of the dependent variable.

#### 5.2.6. Excluding the impacts of contemporaneous policy

In order to accurately identify the impact of NIPDAF policy, it is necessary to exclude the influence of similar policies implemented by CNIPA in the same period.

In 2012, CNIPA launched the national intellectual property pilot demonstration city policy (*IPcity*). From 2012 to 2019, 77 cities and urban areas were selected in seven batches. In 2013, CNIPA implemented the pilot project of patent navigation. From 2013 to 2016, 115 enterprises were approved as national patent operation pilot enterprise (*POfirm*). In 2017, CNIPA implemented the national intellectual property operation service system key construction city policy (*OScity*). From 2017 to 2020, 36 cities were identified in four batches.

The implementation time of these three policies intersects with the NIPDAF policy, and the policy objectives are similar, so they may jointly affect the productivity of enterprises. Therefore, the dummy variables of these three policies are added to the baseline regression separately. Table 6 reports the regression results incorporating contemporaneous policies. It can be seen that the coefficients of the explanatory variable *DID* are still significantly positive. Excluding the impact of contemporaneous policies, the baseline regression results remain robust.

**Table 6:** Results incorporating contemporaneous policies.

	(1)	(2)	(3)
*DID*	0.033***	0.032***	0.032***
	(0.008)	(0.008)	(0.008)
*IPcity*	0.010*		
	(0.006)		
*OScity*		-0.006	
		(0.006)	
*POfirm*			0.043
			(0.038)
*size*	0.164***	0.164***	0.164***
	(0.006)	(0.006)	(0.006)
*roa*	1.061***	1.059***	1.061***
	(0.043)	(0.043)	(0.043)
*grow*	0.120***	0.120***	0.120***
	(0.007)	(0.007)	(0.007)
*lev*	0.066***	0.067***	0.067***
	(0.020)	(0.020)	(0.020)
*labor*	-0.020***	-0.020***	-0.020***
	(0.005)	(0.005)	(0.005)
*board*	-0.105**	-0.107**	-0.104*
	(0.054)	(0.054)	(0.054)
*idr*	0.003	0.003	0.003
	(0.019)	(0.019)	(0.019)
*g_fund*	0.003	0.002	0.003
	(0.004)	(0.004)	(0.004)
*soe*	-0.007	-0.006	-0.006
	(0.014)	(0.014)	(0.014)
*hhi*	-0.019	-0.020	-0.020
	(0.031)	(0.031)	(0.031)
Constant	1.420***	1.423***	1.422***
	(0.120)	(0.120)	(0.120)
*N*	17420	17420	17420
Enterprise FE	Yes	Yes	Yes
Industry FE	Yes	Yes	Yes
Year FE	Yes	Yes	Yes
adj. *R*^2^	0.736	0.736	0.736

### 5.3. Mechanism analysis

Mediation effect test is conducted based on Eqs (2) and (3), and the estimation results are reported in Table 7.

**Table 7:** Results of mechanism analysis.

	(1)	(2)	(3)	(4)	(5)	(6)
	*TI*	*TFP*	*RAE*	*TFP*	*MCA*	*TFP*
*DID*	0.083***	0.032***	-0.005***	0.031***	0.012***	0.023***
	(0.032)	(0.008)	(0.001)	(0.008)	(0.003)	(0.008)
*TI*		0.003				
		(0.002)				
*RAE*				-0.728***		
				(0.050)		
*MCA*						0.868***
						(0.022)
*size*	-0.049**	0.163***	0.003***	0.167***	0.035***	0.135***
	(0.023)	(0.006)	(0.001)	(0.006)	(0.002)	(0.006)
*roa*	0.653***	1.059***	-0.010	1.041***	0.852***	0.324***
	(0.169)	(0.043)	(0.007)	(0.044)	(0.015)	(0.045)
*grow*	-0.046*	0.121***	0.007***	0.129***	0.050***	0.076***
	(0.026)	(0.007)	(0.001)	(0.007)	(0.002)	(0.007)
*lev*	0.006	0.073***	0.007**	0.064***	-0.033***	0.095***
	(0.077)	(0.020)	(0.003)	(0.020)	(0.007)	(0.019)
*labor*	0.107***	-0.022***	-0.004***	-0.024***	-0.013***	-0.010**
	(0.020)	(0.005)	(0.001)	(0.005)	(0.002)	(0.005)
*board*	-0.162	-0.101*	-0.010	-0.124**	-0.009	-0.096*
	(0.211)	(0.054)	(0.009)	(0.055)	(0.019)	(0.051)
*idr*	0.005	0.005	-0.003	-0.004	0.000	0.004
	(0.074)	(0.019)	(0.003)	(0.019)	(0.007)	(0.018)
*g_fund*	0.001	0.000	-0.000	0.000	-0.001***	0.001
	(0.001)	(0.000)	(0.000)	(0.000)	(0.000)	(0.000)
*soe*	0.033	-0.007	-0.003	-0.006	-0.026***	0.011
	(0.055)	(0.014)	(0.002)	(0.014)	(0.005)	(0.013)
*hhi*	-0.189	-0.017	-0.003	-0.020	0.011	-0.017
	(0.121)	(0.031)	(0.005)	(0.032)	(0.011)	(0.030)
Constant	2.042***	1.441***	0.010	1.416***	-0.561***	1.883***
	(0.473)	(0.120)	(0.020)	(0.124)	(0.043)	(0.115)
*N*	17420	17420	17420	17420	17420	17420
Bootstrap	[0.005915, 0.013116] ***					
Enterprise FE	Yes	Yes	Yes	Yes	Yes	Yes
Industry FE	Yes	Yes	Yes	Yes	Yes	Yes
Year FE	Yes	Yes	Yes	Yes	Yes	Yes
adj. *R*^2^	0.799	0.737	0.247	0.739	0.695	0.761

Columns (1) and (2) show the results of the innovation incentive effect of the NIPDAF policy. The coefficients of the independent variable *DID* are both positive and significant at the 1% level. The coefficient of technology innovation (*TI*) is positive but not significant. The NIPDAF policy has a significant promoting effect on firm technological innovation, while the impact of technological innovation on firm productivity is not significant. Thus, further test is conducted using the Bootstrap method. The test results show that the confidence interval for the observed coefficient is [0.005915, 0.013116], excluding 0. This indicates that the mediating effect is significant. Therefore, the NIPDAF policy increases firm productivity by incentivizing technological innovation. H2 is supported.

Columns (3) and (4) present the results of resource allocation effect. The coefficient of the independent variable *DID* in column (3) is -0.005. The coefficient of resource allocation efficiency (*RAE*) in column (4) is -0.728. And both are significant at the 1% level. The NIPDAF policy significantly alleviates the problem of inefficient resource allocation. Inefficient resource allocation reduces enterprise productivity. Therefore, the NIPDAF policy enhances firm productivity by improving resource allocation efficiency. H3 is validated.

Columns (5) and (6) report the results of competitive advantage effect. The impact coefficient of the NIPDAF policy on market competitive advantage (*MCA*) is 0.012, which is significant at the 1% level. The influence coefficient of market competitive advantage (*MCA*) on firm productivity is 0.868, which is significant at the 1% level. Therefore, market competitive advantage plays a significant mediating role in the relationship between the NIPDAF policy and firm productivity. H4 is accepted.

### 5.4. Heterogeneity analysis

In order to further investigate the differential performance of the impact of the NIPDAF policy on productivity in different fields, this study discusses the heterogeneity of regional location, firm nature, and industry. Firstly, there is a huge gap in the level of economic and business environment between different regions in China [25]. The eastern region has relatively high level of economic development, and firms have relatively high awareness and level of intellectual property creation, protection and application. Relatively speaking, the performance of the central and western regions is relatively poor. Secondly, state-owned enterprises (SOEs) have resource advantages, and also facing more flexible regulatory and assessment from the government. In contrast, non-SOEs rely more on intellectual property to expand market and maintain competitive advantage. Therefore, non-SOEs have a stronger motivation to enhance comprehensive intellectual property ability. Thirdly, compared to non-patent intensive industries, patent intensive industries face higher technology competition and greater dependence on intellectual property. Firms in patent intensive industries generally possess independent intellectual property rights and have a high willingness to improve intellectual property ability. Therefore, the sample data is divided based on whether it is a state-owned enterprise, belongs to the eastern or central western regions, and belongs to a patent intensive industry. Patent intensive industries refer to the statistical classification of intellectual property (patents) published by the National Bureau of Statistics of China in 2019. Baseline regression is conducted based on subgroup samples, and the results are shown in Table 8.

**Table 8:** Results of heterogeneity analysis.

	(1)	(2)	(3)	(4)	(5)	(6)
	eastern	central and western	SOEs	non-SOEs	Patent intensive industries	other industries
*DID*	0.044***	0.023	0.016	0.036***	0.028***	0.019
	(0.009)	(0.015)	(0.016)	(0.009)	(0.008)	(0.016)
*size*	0.178***	0.131***	0.145***	0.173***	0.178***	0.148***
	(0.007)	(0.012)	(0.011)	(0.007)	(0.007)	(0.009)
*roa*	0.995***	1.179***	1.249***	1.000***	1.097***	1.061***
	(0.048)	(0.091)	(0.094)	(0.046)	(0.050)	(0.069)
*grow*	0.118***	0.133***	0.123***	0.120***	0.101***	0.136***
	(0.008)	(0.013)	(0.012)	(0.008)	(0.009)	(0.010)
*lev*	0.061***	0.146***	0.060	0.057***	0.131***	-0.002
	(0.022)	(0.041)	(0.039)	(0.022)	(0.023)	(0.032)
*labor*	-0.019***	-0.031***	-0.031***	-0.023***	-0.018***	-0.026***
	(0.006)	(0.011)	(0.009)	(0.006)	(0.007)	(0.007)
*board*	0.004	-0.307***	-0.091	-0.120*	-0.126*	-0.065
	(0.063)	(0.101)	(0.084)	(0.069)	(0.067)	(0.082)
*idr*	0.003	0.016	0.026	-0.013	-0.022	0.023
	(0.022)	(0.036)	(0.032)	(0.023)	(0.023)	(0.029)
*g_fund*	0.000	-0.000	0.001	0.000	0.001	0.001
	(0.000)	(0.001)	(0.001)	(0.000)	(0.000)	(0.001)
*soe*	-0.022	-0.009			-0.004	-0.031
	(0.017)	(0.026)			(0.016)	(0.024)
*hhi*	-0.005	-0.124**	0.006	-0.067*	0.214**	-0.047
	(0.036)	(0.062)	(0.049)	(0.040)	(0.088)	(0.037)
Constant	1.070***	2.280***	1.914***	1.261***	1.085***	1.837***
	(0.140)	(0.242)	(0.231)	(0.139)	(0.150)	(0.194)
*N*	11660	5480	7550	9870	8170	9250
Enterprise FE	Yes	Yes	Yes	Yes	Yes	Yes
Industry FE	Yes	Yes	Yes	Yes	Yes	Yes
Year FE	Yes	Yes	Yes	Yes	Yes	Yes
adj. *R*^2^	0.747	0.749	0.731	0.719	0.766	0.719

Columns (1) and (2) report the regression results of regional heterogeneity. The NIPDAF policy has a significant positive impact on the productivity of firms in the eastern region, with an estimated coefficient of 0.044, which is significant at the 1% level. Correspondingly, the NIPDAF policy has no significant impact on the productivity of firms in the central and western regions. The NIPDAF policy has significant effect in the eastern region, while it has no policy effect in the central and western regions. This may be due to the lack of intellectual property resources and environment in the central and western regions, which hinders the positive impact of the NIPDAF policy on firm productivity.

Columns (3) and (4) represent the regression results of ownership heterogeneity. The impact of NIPDAF policy on the productivity of SOEs is positive but not significant. The impact coefficient of NIPDAF policy on the productivity of non-SOEs is 0.036, and is significant at the 1% level. Compared to SOEs, the NIPDAF policy has a significant promoting effect on the productivity of non-SOEs. This may be because non-SOEs have nimbler intellectual property management strategy and are better at integrating intellectual property resources and other resources to gain competitive advantage, thereby improving firm productivity.

Columns (5) and (6) show the results of industry heterogeneity. The NIPDAF policy has a significant positive impact on the productivity of firms in patent intensive industries, with an impact coefficient of 0.028. Meanwhile, it does not play a significant role in firms in non-patent intensive industries. Therefore, compared to non-patent intensive industries, the NIPDAF policy have a more significant positive effect on patent intensive industries.

## 6. Conclusions and implications

Comprehensive intellectual property ability is crucial to firm productivity. Based on the quasi-natural experiment conducted by the implementation of the NIPDAF policy, using data from 1742 Chinese listed companies from 2011 to 2020 as sample, the time-varying DID method is used to test the impact and mechanism of comprehensive intellectual property ability on firm productivity. The main research conclusions are as follows.

Firstly, the NIPDAF policy significantly increases firm productivity. Compared to non-NIPDAFs, the total factor productivity of NIPDAFs has increased by about 3.3%. This conclusion remains robust even after placebo test, Goodman-Bacon decomposition, PSM-DID method, replacement alternative indicators of dependent variable, and excluding of contemporaneous policies.

Secondly, the NIPDAF policy increases firm productivity by promoting technology innovation, improving resource allocation efficiency, and enhancing market competitiveness.

Thirdly, the impact of the NIPDAF policy exhibits evident heterogeneity. Compared to SOEs, and firms in central and western regions and non-patent intensive industries, the NIPDAF policy has a more significant promoting effect on the productivity of non-SOEs, and firms in the eastern region and patent intensive industries.

Based on the above research conclusions, this paper provides the following insights for developing countries on policies related to firm intellectual property ability. Firstly, leverage policy guidance to guide firms to improve comprehensive intellectual property ability. Enhance the implementation of intellectual property policy, support firm technology innovation and intellectual property acquisition, strengthen judicial protection of intellectual property, and facilitate the transformation of intellectual property. Secondly, create a favorable environment and smooth the channels for comprehensive intellectual property ability. Optimize policy design for firm research and development innovation, resource allocation, and market competition, to help firms further improve productivity. Thirdly, improve the targeted and effective design of policy tools for comprehensive intellectual property ability, and avoid the policy being ineffective for firms in some regions or industries.

This paper empirically investigates the impact of the NIPDAF policy on firm productivity, but there are still certain research limitations. Firstly, this paper focus on the NIPDAF policy. Future research can explore the comprehensive evaluation of intellectual property ability and analyze its impact on firm productivity. Secondly, due to the availability of data, the research sample is listed companies, and the suitability of research conclusions for non-listed companies needs to be verified.
